# Decision-support system for emergency endoscopic surgery in nonvariceal upper gastrointestinal bleeding

**DOI:** 10.1186/s12911-026-03753-1

**Published:** 2026-08-13

**Authors:** Yosuke Kuroda, Nobuhito Ito, Toshihisa Fujiyoshi, Yusuke Satta, Yoji Sasaki, Takeshi Yamamura, Kazuhiro Furukawa, Masanao Nakamura, Hiroki Kawashima, Kenichi Ohara

**Affiliations:** 1 Department of Mechatronics Engineering, Meijo University, Nagoya, Aichi Japan; 2 Department of Endoscopy, Aichi Cancer Center Hospital, Nagoya, Aichi Japan; 3 Japan Red Cross Aichi Medical Center Nagoya Daiichi Hospital, Nagoya, Aichi Japan; 4 Konan Kosei Hospital, Konan, Aichi Japan; 5 Department of Endoscopy, Nagoya University Hospital, Nagoya, Aichi Japan

**Keywords:** Machine learning, XGBoost, Endoscopic intervention, Gastrointestinal bleeding

## Abstract

Nonvariceal upper gastrointestinal bleeding (NVUGIB) is a critical condition requiring timely endoscopic intervention to prevent severe complications. Although widely used, existing risk-scoring systems, such as the Glasgow-Blatchford score (GBS) and Nscore, often exhibit limited predictive accuracy and adaptability across diverse patient populations. This study developed a machine-learning-based clinical decision support system, utilizing tabular data, to predict the need for endoscopic intervention in NVUGIB patients, with the potential to support clinical decision-making and resource utilization, pending further validation. A retrospective dataset comprising 1,989 NVUGIB patients was collected from three Japanese medical institutions between January 2016 and December 2020 and partitioned into training (2016–2019; *n* = 1,467) and validation (2020; *n* = 358) sets. For model development, extreme gradient boosting (XGBoost), a decision-tree-based ensemble algorithm, was employed. The $F_{\beta}$ score was used to optimize thresholds, balancing recall and precision. Model performance was assessed using metrics such as the area under the receiver operating characteristic curve (AUROC), precision, and recall. A comparative analysis was performed against established risk-scoring systems. The XGBoost model achieved an AUROC of 0.822 (95% CI: 0.777–0.863), achieving higher discrimination than Nscore alone (AUROC: 0.776), which was itself included as an input feature, and than GBS (0.576). At a *β = 1.5* threshold, an 89.5% sensitivity and F1-score of 0.723 were achieved. Key predictors, including the blood urea nitrogen to creatinine (BUN/Cr) ratio and Nscore, were identified through feature-importance analysis, demonstrating their substantial contributions to model performance. The XGBoost-based system demonstrated enhanced predictive accuracy regarding the need for endoscopic intervention, suggesting a potential for optimized resource allocation. However, external validation in diverse populations is essential for clinical implementation.

## Clinical trial number

Not applicable.

## Introduction

Nonvariceal upper gastrointestinal bleeding (NVUGIB) encompasses bleeding originating from the esophagus, stomach, and duodenum caused by factors other than varices. Despite a global decline in morbidity and mortality rates in recent years, the prevalence of NVUGIB among elderly patients has been rising [1, 2]. Current Japanese guidelines strongly recommend performing upper gastrointestinal endoscopy within 24 hours after hemodynamic stabilization in high-risk patients with suspected NVUGIB (recommendation grade 1, evidence level A) [3]; international guidelines similarly suggest upper endoscopy within 24 hours of presentation for hospitalized patients with UGIB [4]. To assist in clinical decision-making, scoring systems such as the Glasgow-Blatchford score (GBS) [5] and Rockall Score [6] have been developed to predict the necessity of endoscopic hemostasis and assess the risks of rebleeding and mortality. These tools are particularly effective in identifying low-risk cases with high sensitivity, facilitating outpatient management, and minimizing unnecessary emergency endoscopies. In Japan, population-specific scoring systems, including the Nscore [7], Hirosaki score [8], and Horibe gastrointestinal bleeding score (HARBINGER) [9], have demonstrated superior predictive accuracy than GBS and the Rockall score. Although these models are tailored to clinical data reflecting characteristics common among Japanese patients, their inherent simplicity often limits their capacity to account for diverse patient backgrounds and complex pathologies, highlighting the need for more sophisticated approaches.

Recent NVUGIB studies have increasingly emphasized the development of risk prediction models, utilizing both traditional scoring systems and machine-learning approaches, to enhance clinical outcomes. Unlike conventional scoring systems, machine-learning models can identify complex relationships between features, thereby improving predictive accuracy. For tabular data, various machine-learning algorithms, such as gradient-boosted decision trees (GBDTs) [10, 11], random forest [12], and support vector machine (SVM) [13], have been employed. Among these, Extreme gradient boosting (XGBoost) [14], a variant of GBDTs, has demonstrated high performance, particularly for medical data [15–17]. Although optimal algorithm selection depends on the training data, XGBoost is widely recognized for its ability to handle nonlinear patterns, robustness to noise and outliers, and computational efficiency.

Existing scoring systems, including the (GBS) [5] and Rockall score [6], are designed to predict composite outcomes such as rebleeding, mortality, or hospital stay duration. However, this focus on multiple endpoints often limits their utility in guiding specific interventions such as therapeutic hemostasis [18]. Similarly, machine-learning-based risk prediction models have predominantly targeted composite outcomes, further underscoring the need for approaches that focus on specific clinical interventions to improve decision-making and patient care [15, 19]. Although these predictive systems have demonstrated strong performance for composite outcomes, they often lack granularity for predicting specific interventions such as endoscopic hemostasis. Furthermore, traditional machine-learning models typically classify patients using fixed cutoff values. However, risk prediction requires the reliable identification of high-risk patients as positive. Addressing this requires dynamically adjusting cutoff values to align with the output distribution of the machine-learning model.

This study primarily aimed to develop a predictive model using XGBoost to determine the necessity of emergency endoscopic intervention for patients with NVUGIB. By incorporating diverse features—many underutilized in conventional scoring systems—the study aimed to achieve higher predictive accuracy than existing models. Moreover, it attempted to design a system practically implementable in clinical settings to support the triage and prioritization of patients for urgent endoscopy, rather than to reduce the total number of endoscopies. Specifically, this study makes the following key contributions:

- It specifically focuses on hemostatic intervention as the primary outcome.
- It develops and employs a moderately sized multicenter dataset comprising approximately 2,000 patients from Japan.
- It introduces a dynamic threshold adjustment method to enhance clinical adaptability

The remainder of this paper is structured as follows: Section “Methods” details the methods used for predictive model development, including data collection, preprocessing, and the machine-learning process. Section “Results” presents the performance evaluation results, including comparisons with existing risk-scoring systems and feature-importance analysis. Section “Discussion” discusses the implications of the findings, interprets the results within the context of clinical applications, and acknowledges the study limitations. Finally, Section “Conclusion” concludes the study, summarizing the contributions and suggesting directions for future research and real-world implementation.

## Methods

### Machine-learning process

**Fig. 1:**
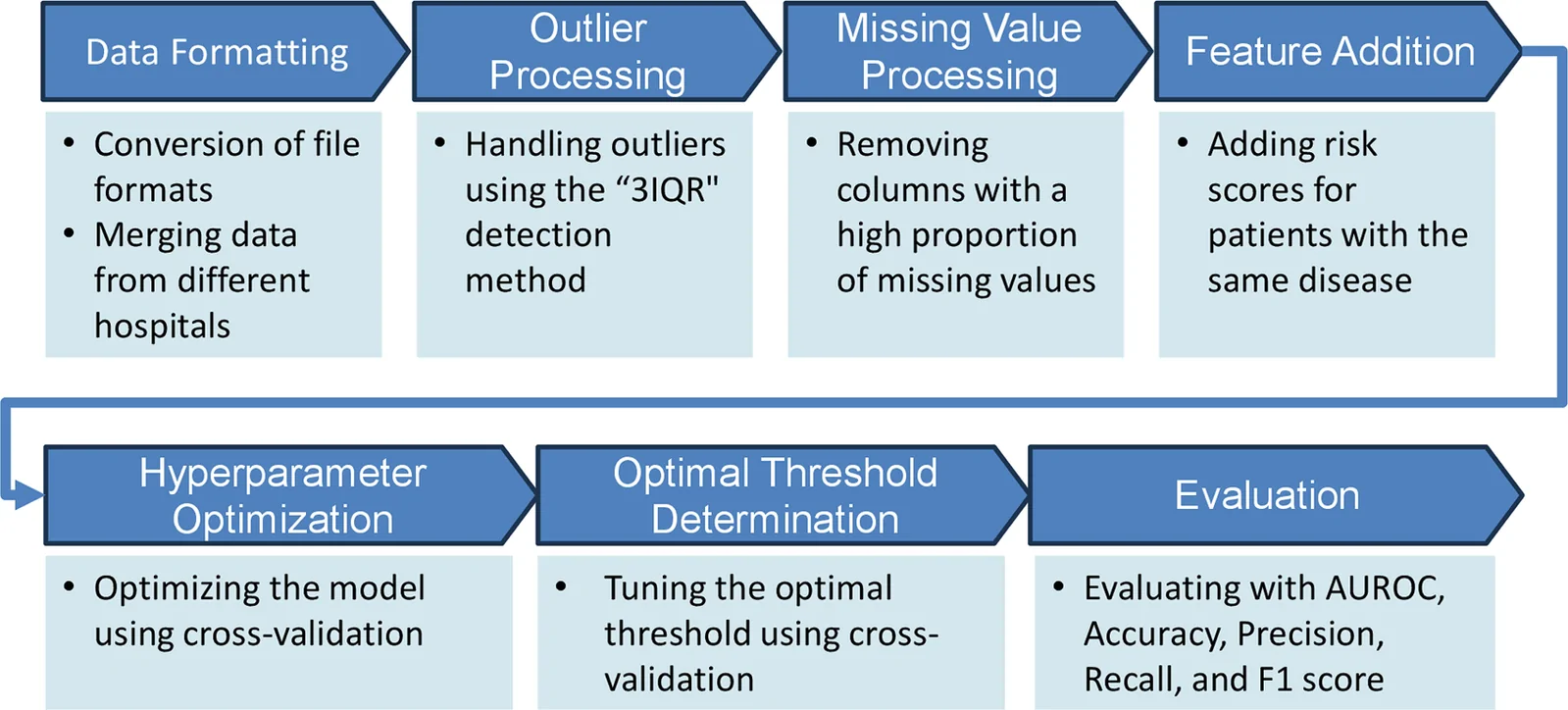
Machine-learning process employed in this study

This section describes the machine-learning process employed in this study, from data collection to performance evaluation. Figure 1 illustrates the entire process, wherein the upper portion illustrates data preprocessing, whereas the lower portion depicts model construction and evaluation.

### Data collection

Data for this study were collected retrospectively from three institutions—Nagoya University Hospital, Japanese Red Cross Nagoya Daiichi Hospital, and Konan Kosei Hospital—between January 2016 and December 2020. A total of 1,989 consecutive patients with suspected NVUGIB were included. “Suspected NVUGIB” was defined as patients presenting with one or more of the following clinical findings: hematemesis, melena, coffee-ground vomitus, acute hemoglobin drop, or hemodynamic instability suggestive of upper gastrointestinal bleeding. After endoscopic evaluation, all included cases were confirmed to have nonvariceal upper gastrointestinal bleeding. No patients with variceal bleeding or non-GI bleeding sources were included in the final dataset.

In this study, endoscopic intervention refers to therapeutic endoscopic hemostasis (e.g., clipping, thermal coagulation, or injection therapy) performed during the index emergency endoscopy, which served as the target (positive) outcome for prediction. The primary endpoint (need for endoscopic intervention) was determined from the endoscopy report and medical records of the index emergency endoscopy: a case was labeled positive if therapeutic hemostasis (e.g., clipping, thermal coagulation, or injection therapy) was performed by the attending endoscopist, and negative otherwise. The endoscopist’s determination at the index procedure thus served as the reference standard.

The intended clinical scenario for this model is the triage of patients in whom variceal bleeding has been clinically excluded or considered unlikely prior to endoscopy. In practice, variceal bleeding can often be clinically suspected based on the presence of liver cirrhosis, a history of esophageal or gastric varices, or relevant imaging findings indicating portal hypertension; such patients typically undergo emergency endoscopy without delay, irrespective of risk stratification. For the remaining patients presenting with suspected NVUGIB, unnecessary emergency endoscopy imposes a substantial burden on after-hours physicians and may disproportionately affect institutions in regions with physician shortages. Given that approximately 40% of the enrolled patients required endoscopic intervention, identifying high-risk individuals who genuinely require urgent endoscopy represents a clinically meaningful application of this model.

The dataset comprised the following features:

- Sex
- Age
- Systolic blood pressure (SBP)
- Diastolic blood pressure (DBP)
- Pulse rate
- Hemoglobin (Hb)
- Albumin (Alb)
- Lymphocyte count
- Glomerular filtration rate (GFR)
- BUN
- Cr
- Presence of syncope
- Hematemesis
- Coffee ground vomitus
- Melena
- Liver disease
- Heart failure
- Use of antiplatelet agent
- Use of anticoagulant agent
- Use of proton pump inhibitors (PPIs)
- History of blood transfusion
- Presence of tumors
- Shock index
- BUN/Cr ratio

Table 1 summarizes the distribution of these features across the entire dataset, as well as for the training (2016–2019; *n* = 1467) and test (2020; *n* = 358) sets. Binary variables, including sex and clinical symptoms, are presented as counts, whereas continuous variables, encompassing vital signs and laboratory values, are expressed as means *±* standard deviations. The intervention outcome, serving as the target variable, is also included. These statistics offer an overview of dataset composition and highlight potential differences between the training and test sets.

**Table 1:** Summary statistics of features for the overall, training, and test datasets

	Variable	All (*n=1825*)	Train (*n=1467*)	Test (*n=358*)
Binary (No / Yes)	Sex (Female / Male)	677 / 1148	554 / 913	123 / 235
	Syncope	1706 / 119	1377 / 90	329 / 29
	Hematemesis	1375 / 450	1118 / 349	257 / 101
	Coffee-ground vomitus	1541 / 284	1232 / 235	309 / 49
	Melena	636 / 1189	507 / 960	129 / 229
	Liver disease	1750 / 75	1411 / 56	339 / 19
	Heart failure	1590 / 235	1273 / 194	317 / 41
	Antiplatelet agent${}^{{\mathrm{a}}}$	1481 / 343	1211 / 256	270 / 87
	Anticoagulant agent	1587 / 238	1273 / 194	314 / 44
	PPI${}^{{\mathrm{a}}}$	1296 / 528	1062 / 404	234 / 124
	Blood transfusion	1079 / 746	892 / 575	187 / 171
	Cancer${}^{{\mathrm{a}}}$	1489 / 331	1210 / 253	279 / 78
Continuous	Age (years)	*71.0 ± 15.4*	*70.6 ± 15.4*	*72.4 ± 15.2*
	SBP (mmHg)	*119 ± 26.1*	*119 ± 26.1*	*120 ± 25.7*
	DBP (mmHg)	*68.9 ± 17.1*	*68.8 ± 17.3*	*69.4 ± 16.6*
	Pulse (/min)	*91.8 ± 19.6*	*91.9 ± 19.4*	*91.3 ± 20.3*
	Hemoglobin (g/dL)	*8.98 ± 2.93*	*9.06 ± 2.92*	*8.64 ± 2.91*
	Albumin (g/dL)	*3.19 ± 0.68*	*3.23 ± 0.67*	*3.05 ± 0.71*
	Lymphocyte ($/\mu\textrm{L}$)	*1540 ± 940*	*1530 ± 925*	*1560 ± 1000*
	GFR ($\textrm{mL/min/}1.73\,m^2$)	*62.1 ± 38.2*	*64.0 ± 39.0*	*54.1 ± 33.5*
	BUN (mg/dL)	*35.1 ± 21.8*	*33.9 ± 20.2*	*40.2 ± 26.8*
	Cr (mg/dL)	*1.11 ± 0.95*	*1.00 ± 0.47*	*1.58 ± 1.86*
	BUN/Cr ratio	*35.4 ± 22.6*	*36.1 ± 23.8*	*32.5 ± 16.5*
	Shock index	*0.81 ± 0.27*	*0.81 ± 0.26*	*0.81 ± 0.29*
Clinical risk score (points)	GBS	*9.17 ± 4.18*	*9.06 ± 4.23*	*9.64 ± 3.94*
	Nscore	*1.90 ± 1.47*	*1.86 ± 1.45*	*2.04 ± 1.52*
	Modified Nscore	*1.69 ± 1.47*	*1.70 ± 1.49*	*1.64 ± 1.42*
	Hirosaki score	*1.92 ± 2.27*	*1.93 ± 2.26*	*1.87 ± 2.32*
	HARBINGER	*1.46 ± 0.85*	*1.49 ± 0.83*	*1.36 ± 0.91*
Target	Endoscopic intervention (No / Yes)	1097 / 728	892 / 575	205 / 153

Continuous variables are expressed as mean *±* standard deviation; binary variables are shown as counts (no / yes). Units are given in parentheses

^a^ Missing values: antiplatelet agent (*n* = 1), PPI (*n* = 1), cancer (*n* = 5); counts are based on available data.Abbreviations: SBP, systolic blood pressure; DBP, diastolic blood pressure; GFR, glomerular filtration rate; BUN, blood urea nitrogen; Cr, creatinine; PPI, proton pump inhibitor; GBS, Glasgow–Blatchford score; HARBINGER, Horibe GI bleeding score

### Data partition

**Fig. 2:**
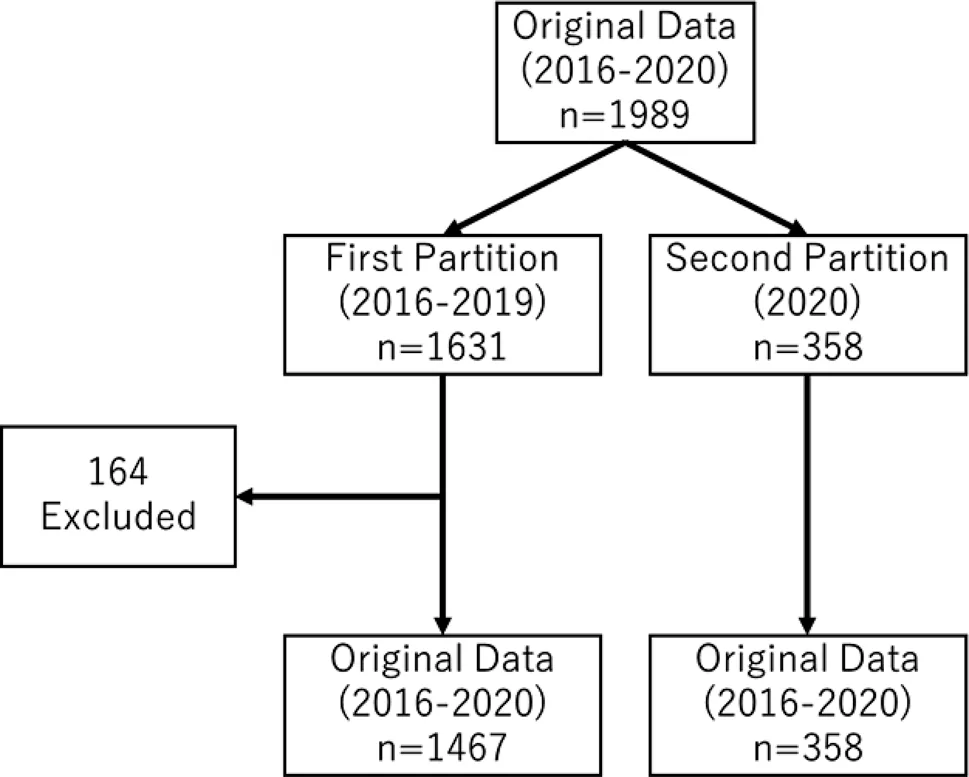
Data partitioning process

Figure 2 shows the data partitioning process. The dataset was partitioned into two time-series subsets: one covering 2016–2019 and the other encompassing 2020. The 2016–2019 data (*n* = 1631) underwent outlier processing and served as the training set. After excluding 164 entries identified as outliers, the final training set comprised 1467 entries. The 2020 data (*n* = 358) were not subjected to outlier processing or intentional modification and reserved solely for accuracy evaluation.

### Data preprocessing

This study incorporated additional features derived from existing risk-scoring systems, including the GBS, Nscore, modified Nscore, Hirosaki score, and HARBINGER [7–9, 20]. Outliers were processed to mitigate the influence of erroneous or extreme values (e.g., transcription errors).

These established risk scores (GBS, Nscore, modified Nscore, Hirosaki score, and HARBINGER) were included as input features of the XGBoost model. Consequently, the model should be understood as building upon these scores—particularly Nscore—rather than as an independent competitor to them, and the comparisons presented below reflect the incremental value of adding further routinely available variables to these scores.

Outlier detection employed the interquartile range (IQR) method [21]. Specifically, the first (*Q1*) and third (*Q3*) quartiles were calculated for each feature. Upper and lower thresholds were defined as follows:

$$\eqalign{& {\rm{Upper Threshold}} = Q3 + 3 \times {\rm{IQR}}, \cr & {\rm{Lower Threshold}} = Q1 - 3 \times {\rm{IQR}} \cr} $$

A conservative multiplier of $3 \times\textrm{IQR}$ (Tukey’s “far out” fences) was chosen, rather than the more common $1.5 \times\textrm{IQR}$, so that only the most extreme and physiologically implausible values—most likely attributable to transcription or measurement error—were removed, while the majority of clinically genuine extreme values were retained. Outlier processing was applied only to the training set; the test set was left entirely unprocessed, so that all reported performance metrics reflect unfiltered, real-world data.

Any record containing at least one value outside this range was considered an outlier. As a result, 164 records from the 2016—2019 dataset were excluded from the training set. Importantly, all outlier removal was performed only on the training data to avoid information leakage.

Missing data were minimal: only the antiplatelet agent (*n* = 1; 0.05%), PPI (*n* = 1; 0.05%), and cancer (*n* = 5; 0.27%) variables contained missing values, and no variable exceeded 0.3% missingness (well below the 30% threshold for column removal). Missing values were not imputed but were handled natively by XGBoost, which assigns a learned default direction for missing entries at each tree split.

### Machine-learning algorithm

XGBoost was employed owing to its suitability for tabular datasets [14]. XGBoost, a decision-tree-based machine-learning algorithm, is recognized for its high prediction accuracy and computational efficiency and has been widely adopted across various research domains, including medicine.

During training, XGBoost generates decision trees based on hyperparameters influencing model performance. Hyperparameter tuning was conducted using Optuna, a hyperparameter optimization framework that automates the search for optimal hyperparameters [22].

The key hyperparameters included max_depth, which determines the maximum depth of each decision tree. The hyperparameter ranges, detailed in Table 2, were optimized using Optuna. To address the class imbalance, the “scale_pos_weight” parameter was adjusted to match the ratio of positive to negative samples in the training dataset for each iteration.

**Table 2:** Hyperparameter tuning ranges for XGBoost optimization

Hyperparameter	Lower Limit	Upper Limit
max_depth	3	10
min_child_weight	1	10
subsample	0.5	1.0
gamma	0	1.0
reg_alpha	$1 \times 10^{-5}$	100
reg_lambda	$1 \times 10^{-5}$	100
colsample_bytree	0.5	1.0
max_delta_step	0.5	1.0

### Optimal-threshold tuning

Recall is often paramount in medical classification tasks. The $F_{\beta}$ score is employed to optimize the threshold for imbalanced classifications, enabling users to arbitrarily adjust the balance between recall and precision, tailoring risk prediction to specific needs.

This study adopted stratified 10-fold cross-validation to evaluate the generalizability of the model while searching for the threshold that maximizes the $F_{\beta}$ score. Specifically, the threshold maximizing the $F_{\beta}$ for a given *β* was determined for each fold, and the average value was designated as the optimal threshold for that *β*. The $F_{\beta}$ score is calculated as follows:

$$F_{\beta} = (1 + \beta^2) \cdot \frac{\mathrm{Precision} \cdot \mathrm{Recall}}{(\beta^2 \cdot \mathrm{Precision}) + \mathrm{Recall}}$$

where precision and recall are defined as:

$$\mathrm{Precision} = \frac{\mathrm{TP}}{\mathrm{TP} + \mathrm{FP}}, \quad\mathrm{Recall} = \frac{\mathrm{TP}}{\mathrm{TP} + \mathrm{FN}}$$

True positives (TPs), false positives (FPs), and false negatives (FNs) are defined as follows:

- **TP**: Number of positive cases correctly predicted by the model.
- **FP**: Number of negative cases incorrectly predicted as positive.
- **FN**: Number of positive cases incorrectly predicted as negative.

By adjusting the *β* parameter, the trade-off between precision and recall can be controlled. For example, when *β = 1*, the $F_{\beta}$ score reduces to the F1-score, weighting precision and recall equally. Conversely, larger *β* values emphasize recall, making it suitable for applications where minimizing FNs is critical.

*β* value selection should also be adjusted dynamically based on disease severity and test cost. For example, when screening for highly lethal diseases, a strategy of setting $\beta \ge 2$ and prioritizing recall is effective. Implementing this method enables customization according to the practice policies of medical institutions, contributing to personalized medicine.

### Validation

The performance of the developed machine-learning model was verified using test data independent of the training process, employing several evaluation metrics, including the area under the receiving operator characteristic curve (AUROC), accuracy, recall, and precision. The effectiveness of the machine-learning-based predictive model was confirmed by comparing it with that of existing clinical scoring systems. Additionally, the high sensitivity of the prediction was validated by adjusting the optimal thresholds.

### Feature importance identification using SHapley additive exPlanations (SHAP)

SHAP interprets the XGBoost model by quantifying each feature’s contribution to predictions [23], thereby enhancing model transparency and aiding clinical decision-making. SHAP values, grounded in cooperative game theory, measure the marginal contribution of each feature, thus facilitating fair allocation of importance and clarifying feature impact. This study employed SHAP to identify key predictors of endoscopic intervention in patients with NVUGIB, enhancing model interpretability and reliability.

## Results

### Predictive performance of XGBoost

The predictive performance of the XGBoost model was evaluated using key metrics: AUROC, accuracy, sensitivity (recall), specificity, precision, and F1 score. Bootstrap percentile methods were employed to estimate confidence intervals (CIs) for these metrics [24, 25]. Specifically, 10,000 bootstrap samples were drawn with replacements from the original dataset, and the 2.5th and 97.5th percentiles of the bootstrap distribution were calculated to obtain the 95% CI. Table 3 summarizes these results. The XGBoost model achieved high AUROC values and a balanced trade-off between sensitivity and specificity. Specifically, it achieved an average AUROC of 0.822 (95% CI: 0.777–0.863), accuracy of 0.749 (95% CI: 0.704–0.793), recall of 0.752 (95% CI: 0.680–0.818), precision of 0.689 (95% CI: 0.616–0.760), and F1 score of 0.719 (95% CI: 0.660–0.772). These results indicate that the XGBoost model effectively classified positive and negative cases.

**Table 3:** Comparison of the XGBoost model with traditional risk-scoring systems

Model	Cutoff	AUROC	Accuracy	Precision	Recall	F1
XGBoost (default, 0.5)	*≥ 0.500*	**0.822 (0.777–0.863)**	**0.749**	0.689	0.752	0.719
XGBoost ($F_{0.5}$)	*≥ 0.638*	–	0.743	**0.785**	0.549	0.646
XGBoost ($F_{1}$)	*≥ 0.406*	–	0.721	0.635	0.817	0.714
XGBoost ($F_{1.5}$)	*≥ 0.322*	–	0.707	0.606	0.895	**0.723**
XGBoost ($F_{2}$)	*≥ 0.265*	–	0.679	0.578	0.922	0.710
XGBoost ($F_{2.5}$)	*≥ 0.177*	–	0.587	0.508	0.980	0.670
XGBoost ($F_{3}$)	*≥ 0.131*	–	0.534	0.478	0.987	0.644
Nscore	*≥ 2*	0.776 (0.728–0.821)	0.704	0.609	0.856	0.712
Hirosaki score	*≥ 1*	0.728 (0.676–0.777)	0.589	0.511	0.902	0.652
HARBINGER	*≥ 1*	0.658 (0.603–0.711)	0.506	0.459	0.882	0.604
GBS	*≥ 7*	0.576 (0.518–0.635)	0.478	0.438	0.791	0.564
GBS	*≥ 1*	–	0.436	0.431	0.993	0.601

For the XGBoost model, several operating points are shown: the default probability cutoff (0.5) and thresholds optimized for different $F_{\beta}$ scores. The AUROC is threshold-independent and is therefore identical across all XGBoost operating points. Values in parentheses are 95% confidence intervals; the best value in each metric column is in bold

Abbreviations: AUROC, area under the receiver operating characteristic curve; GBS, Glasgow–Blatchford score; HARBINGER, Horibe GI bleeding score

### Comparison with risk-scoring systems

The performance of the XGBoost model was compared with that of several established risk-scoring systems commonly used in clinical practice: Nscore, Hirosaki score, HARBINGER, and GBS. Cutoff values were determined by referring to relevant studies [5, 7–9, 26]. Table 3 presents a comparative analysis of the key metrics.

The XGBoost model performed better than traditional risk-scoring systems across multiple evaluation criteria. For example, with *β=0.5*, it achieved the highest AUROC value of 0.822 (95% CI: 0.777–0.863), which showed higher discrimination than that of Nscore (AUROC: 0.776 (95% CI: 0.728–0.821)) and Hirosaki score (AUROC: 0.728 (95% CI: 0.676–0.777)). Additionally, the F1-score of the XGBoost model with *β=1.5* reached 0.723, indicating a well-balanced trade-off between precision and recall.

The cutoffs for the traditional scoring systems were adopted from their original publications, whereas the XGBoost threshold was determined by optimizing the $F_{\beta}$ score on the training data. Because these threshold-selection criteria differ, the threshold-dependent metrics (accuracy, precision, recall, and F1-score) should be interpreted as indicative rather than directly comparable across models. The AUROC, which is threshold-independent, serves as the primary metric for comparison.

To formally assess whether the higher AUROC of the XGBoost model was statistically significant, we performed the DeLong test on the test set (*n* = 358), comparing the XGBoost model with each scoring system. The XGBoost model showed significantly higher discrimination than all evaluated scores, with the AUROC difference (*Δ*) reaching statistical significance: Nscore (*Δ = 0.046*; 95% CI, 0.012–0.080; *p = 0.009*), Hirosaki score (*Δ = 0.094*; 95% CI, 0.045–0.143; *p < 0.001*), HARBINGER (*Δ = 0.164*; 95% CI, 0.117–0.211; *p < 0.001*), and GBS (*Δ = 0.245*; 95% CI, 0.180–0.311; *p < 0.001*).

The markedly lower AUROC of the GBS in our cohort should not be interpreted as inferiority of the score itself. The GBS is a triage tool intended for the full spectrum of patients with suspected UGIB, whereas our cohort is restricted to already-endoscoped, uniformly high-risk patients (mean GBS 9.17); this compression of its dynamic range (spectrum bias) attenuates its apparent discrimination, and in unselected populations the GBS achieves substantially higher discrimination for predicting endoscopic therapy [26]. Moreover, the GBS tends to show lower discrimination in Japanese NVUGIB cohorts than in Western populations: Japanese multicenter studies have reported GBS AUROCs of approximately 0.63 and 0.68 for predicting the need for endoscopic intervention [9, 27]. The value observed in our cohort is therefore consistent with both this population difference and the spectrum bias described above.

### Feature importance analysis using SHAP values

**Fig. 3:**
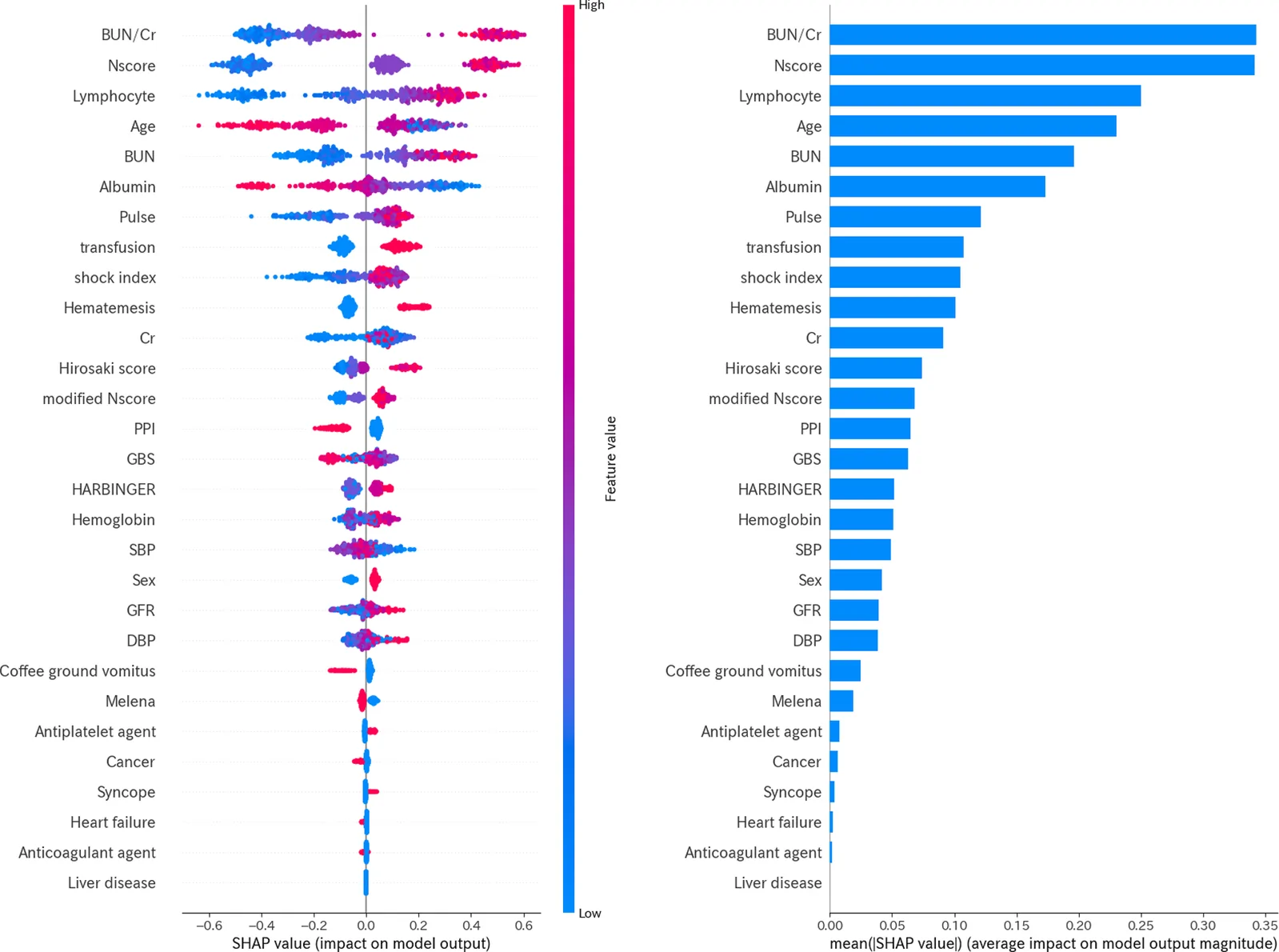
SHAP-based analysis of feature importance

The contribution of each feature to the predictions of the XGBoost model was analyzed using SHAP values (Fig. 3). The left panel depicts the distribution of SHAP values for individual features, whereas the right panel ranks features by their average contribution to predictions.

Key predictors included the BUN/Cr ratio and Nscore, which exhibited the highest mean absolute SHAP values, indicating their substantial influence on model performance. Other important features were lymphocyte count, age, BUN, and albumin.

Conversely, underlying disease and medication history—such as antiplatelet use, cancer, syncope, heart failure, anticoagulants, and liver disease—had relatively minor impacts on predictions, with mean absolute SHAP values below 0.05.

Among traditional scoring systems, Nscore contributed most significantly to predictions, followed by the Hirosaki score, modified Nscore, GBS, and HARBINGER.

## Discussion

### Summary of key findings

The results demonstrated that the XGBoost model outperformed existing scoring systems (Nscore, Hirosaki score, HARBINGER, and GBS) for predicting the necessity of endoscopic intervention. Specifically, the XGBoost model achieved a high AUROC of 0.822 (95% CI: 0.777–0.863), indicating well-balanced predictive accuracy between sensitivity and specificity. Moreover, its dynamic cutoff adjustment feature enabled flexible applications tailored to specific clinical needs. Furthermore, SHAP analysis identified the BUN/Cr ratio as the most important feature, whereas Nscore was the most significant among the existing scoring systems. Because the established scores, including Nscore, are themselves inputs to the model, the improvement over Nscore should be interpreted as the incremental discrimination gained by combining Nscore with additional routinely available variables, rather than as evidence that the model outperforms an independent Nscore.

The intended benefit of the model is not a reduction in the number of endoscopies, since diagnostic endoscopy remains necessary irrespective of the prediction and the recommended high-recall thresholds flag more rather than fewer patients. Rather, the model is intended to support triage and prioritization: identifying patients who most likely require therapeutic endoscopy and should be prioritized for urgent (after-hours) intervention, while others may be safely deferred to a routine daytime procedure.

### Interpretation of results

The superior predictive performance of the XGBoost model can be attributed to its ability to effectively capture complex interactions among features not considered by traditional scoring systems. For example, the model achieved high predictive accuracy by integrating biochemical indicators such as the BUN/Cr ratio and albumin levels with patient background information such as age and lymphocyte count.

These findings align with those of previous studies [28–30]. While the association of BUN/Cr ratio and albumin levels with the severity of gastrointestinal bleeding is well-established, this study confirmed their crucial role in predictive modeling. The contribution of lymphocyte count is consistent with a growing body of work on lymphocyte-based inflammatory markers in UGIB—particularly the neutrophil-to-lymphocyte ratio, which has been associated with mortality, the need for endoscopic treatment, and transfusion [31, 32]—and warrants further study in predictive modeling. Conversely, baseline diseases and medication history (e.g., antiplatelet or anticoagulant drugs) had a limited impact, providing new insights into risk-assessment methods for clinical practice.

Additionally, the findings suggested potential regional specificity in data characteristics. Specifically, scoring systems developed in Japan, such as Nscore and Hirosaki score, were ranked higher than GBS, which is widely used internationally. This indicates that dataset characteristics unique to Japan may have influenced the results.

### Limitations

Several limitations should be considered when interpreting the results and assessing their significance.

First, the dataset was derived from three hospitals within the same region of Japan, which may limit generalizability to other regions or healthcare systems with different patient characteristics and practice patterns. In addition, the study included only patients who underwent emergency endoscopy, potentially enriching the dataset with more severe cases and limiting the applicability of the model to milder presentations. External validation using larger and more diverse cohorts is necessary.

Second, this study included only cases involving emergency endoscopy. This criterion may have resulted in an overrepresentation of severe cases requiring urgent intervention, introducing a potential selection bias. Consequently, the performance of the model for less severe cases, or those not requiring emergency procedures, may not have been adequately captured.

Third, owing to data availability, only four scoring systems (Nscore, Hirosaki score, HARBINGER, and GBS) were evaluated. Other widely used systems, such as the Rockall and Baylor bleeding scores, were excluded. Incorporating features from these excluded scoring systems, which are not included in the current dataset, may enhance the accuracy of the model.

Fourth, although the DeLong test confirmed that the XGBoost model achieved significantly higher discrimination than the existing scores, the threshold-dependent metrics (accuracy, precision, recall, and F1-score) were obtained using different criteria across models and are therefore not directly comparable. Future studies should adopt a unified threshold-determination framework to enable fair head-to-head comparisons.

Fifth, the temporal data split used in this study (training: 2016–2019; test: 2020) may introduce temporal bias, as changes in clinical practice or patient characteristics over time could affect model performance.

Sixth, the Nscore was derived by several of the present co-authors using data from one of the participating institutions, and its derivation cohort partially overlaps with our training data in period and patients. This shared provenance may favor the Nscore in the present comparison; the incremental value of the model over Nscore should therefore be interpreted accordingly and confirmed in fully independent cohorts.

Seventh, we assessed only discrimination and threshold-dependent operating characteristics; the calibration of the predicted probabilities and the net clinical benefit (e.g., by decision-curve analysis) were not evaluated. Calibration and decision-curve analyses are therefore required to substantiate the clinical-utility and resource-optimization claims before the model can be deployed at a fixed threshold.

Eighth, outliers were removed from the training data using a $3\times\textrm{IQR}$ criterion. Although this conservative threshold was intended to exclude only implausible values, some excluded extreme values (e.g., very high BUN or creatinine, very low hemoglobin, or high shock index) may represent genuine, prognostically important physiology rather than errors; their removal could attenuate the model’s ability to identify the most severe cases. A sensitivity analysis without outlier removal, and adjudication of excluded records as data-entry errors versus plausible physiology, were not performed and remain important directions for future work.

Ninth, the risk-scoring systems—particularly the GBS—were evaluated within a cohort restricted to patients who had already undergone emergency endoscopy. Because the GBS is intended for triage across the full spectrum of suspected UGIB, its discrimination is attenuated in this uniformly high-risk population (spectrum/selection bias); its low AUROC here therefore should not be interpreted as evidence of our model’s superiority.

### Future directions

Advancing this research requires several key efforts. First, large-scale, multicenter studies are essential to validate the performance of the model across diverse patient populations and healthcare settings to confirm its generalizability. This would allow evaluating its applicability in different regions and medical systems, thereby enhancing its reliability. Second, enhancing model transparency through explainable AI (XAI) techniques is imperative. Because XGBoost is inherently a black-box model, interpreting its decision-making process remains challenging. While SHAP provides valuable insights, multicollinearity among explanatory variables can lead to misinterpretation of feature importance. This can be addressed by integrating alternative interpretability methods, such as local interpretable model-agnostic explanations, which offer complementary insights and a clearer understanding of the model’s decision-making process [33]. Furthermore, feature-selection techniques that maintain predictive performance while improving interpretability can increase trust and acceptance, particularly in clinical settings.

Finally, prospective trials are required to demonstrate the clinical utility of the predictive model in real-world scenarios. These trials should integrate the predictive model into actual clinical workflows and evaluate its impact on patient outcomes. This would provide robust evidence of its effectiveness and represent a significant step toward its implementation in medical practice.

## Conclusion

This study developed an XGBoost-based machine-learning model to predict the necessity of endoscopic intervention in patients with NVUGIB. Using a dataset of 1,989 patients from three Japanese institutions, the model demonstrated superior predictive performance (AUROC: 0.822) than conventional risk-scoring systems. Additionally, a dynamic threshold adjustment method based on the $F_{\beta}$ score was introduced, confirming its adaptability in clinical settings.

These findings highlight the potential of machine learning as a clinical decision support system that may support triage and resource utilization, although these potential benefits require prospective validation. However, several limitations exist. Because the data were collected from only three institutions in Japan, further validation is required to ensure generalizability across different regions and healthcare settings. Additionally, the interpretability of the XGBoost model remains a challenge, necessitating efforts to enhance transparency and explainability.

Future research should focus on large-scale, multicenter validation; integration of XAI techniques; and real-world clinical implementation. With these advancements, the model could achieve widespread adoption in medical practice, contributing to more efficient and data-driven decision-making for endoscopic management.

## Data Availability

The data supporting the findings of this study are available from the authors upon request. However, due to ethical restrictions imposed by Nagoya University Hospital these data are not publicly available.
